# Evaluation of Crack Resistance Performance of Semi-Flexible Pavement Materials

**DOI:** 10.3390/ma18122796

**Published:** 2025-06-13

**Authors:** Songqiang Chen, Jianfei Zheng, Xi Wu, Lufan Li

**Affiliations:** 1 Department of Civil Engineering, School of Engineering, Hangzhou City University, Hangzhou 310015, China; wuxi@hzcu.edu.cn; 2 Zhejiang Engineering Research Center of Intelligent Urban Infrastructure, Hangzhou City University, Hangzhou 310015, China; 3 Huadong Engineering Corporation Limited, Hangzhou 310015, China; zheng_jf@hdec.com

**Keywords:** semi-flexible pavement, material properties, pavement structure, cracking resistance

## Abstract

Semi-flexible pavement (SFP) materials have garnered extensive application and research attention owing to their exceptional deformation resistance. The crack resistance of SFP materials constitutes a critical aspect of their road performance. This study conducts a comprehensive analysis of the crack resistance of SFP materials through material characterization and structural mechanical response evaluation. To assess the cracking behavior of SFP materials across the entire temperature spectrum, three experimental methodologies were employed: low-temperature flexural tensile testing, indirect tensile testing, and semi-circular bending tensile testing. Experimental findings reveal that SFP materials exhibit superior crack resistance compared to SMA-13 under ambient and elevated temperature conditions, while demonstrating inferior performance relative to SMA-13 in low-temperature environments. Through a comparative analysis of structural mechanical responses between SMA-13 and SFP pavements, it was determined that the implementation of a single-layer SFP material can reduce pavement tensile strain by 30–50%. This investigation provides comprehensive insights into the crack resistance characteristics of SFP materials and offers valuable guidance for material selection in pavement structural design.

## 1. Introduction

Semi-flexible pavement (SFP) is a composite material formed by injecting a high-fluidity cement-based grout into a large-pore asphalt mixture. Initially developed in the early 1960s in France and Japan, SFP was subsequently adopted in the United States (US) and the United Kingdom (UK) [1,2,3]. Combining the flexibility of asphalt pavements with the rigidity of cementitious materials, SFP has been extensively applied in areas prone to severe rutting distress, such as intersections, long longitudinal slopes, and heavy-duty pavements [4,5,6]. The superior mechanical strength of cement-based materials, which is temperature-independent compared to asphalt mixtures, enables SFP to effectively mitigate rutting issues caused by high temperatures and heavy traffic loads [7,8]. Additionally, SFP typically requires a curing period of 2–4 h, rendering it highly suitable for urban pavement maintenance applications due to its rapid construction turnaround [9]. Furthermore, while the cement-based grout fills the voids of the asphalt skeleton, vehicular tires remain in direct contact with the asphalt matrix, ensuring that the SFP retains the driving comfort characteristic of conventional asphalt pavements.

Recent studies have demonstrated growing research interest in semi-flexible pavement (SFP). Husain et al. investigated the statistically significant differences in volumetric properties, durability, and mechanical strength of SFP produced using three distinct aggregate gradations specified by the Road Engineering Association of Malaysia (REAM) [10]. Liu et al. conducted a comprehensive investigation into the formulation and performance of powdered polysilicate grouting materials for semi-flexible pavements; however, regrettably, their study omitted an in-depth analysis of rheological characteristics [11]. In their experimental protocol, a water-reducing admixture was incorporated to enhance the flowability of the grouting material. In contrast, Xiang et al. demonstrated that the addition of limestone powder to an alkali-activated slag–fly ash system effectively reduced the yield stress and plastic viscosity of the slurry [12]. Furthermore, the incorporation of finer sand powder in alkali-activated metakaolin-based grouts was found to significantly improve rheological properties while concurrently mitigating shrinkage by approximately 30% [13]. Zhao et al. analyzed the influence of five gradation types on the volumetric stability, microstructural characteristics, high-temperature performance, moisture resistance, and strength properties of SFP. Their findings suggested that continuous gradation exhibits superior applicability in regions with significant seasonal temperature variations but limited rainfall, whereas single-sized gradation is more suitable for areas with minimal temperature fluctuations and high summer temperatures accompanied by heavy precipitation [14]. The gradation design primarily governs the porosity of the asphalt skeleton, which typically requires an air void content of 20–30%. Concurrently, grouting materials—characterized by high fluidity, rapid curing, and adequate mechanical strength—have emerged as another critical research focus, with their key properties summarized in Table 1. Wang et al. demonstrated that the incorporation of carboxyl latex enhances multiple performance metrics, including high-temperature rutting resistance, low-temperature crack resistance, moisture damage resistance, and fatigue durability [15]. Koting et al. explored the effects of polymer types and dosages in high-performance water reducers (specifically two polycarboxylate ether-based polymers and a sulfonated naphthalene formaldehyde condensate) on the rheological properties of cementitious grouts [16]. Zhang et al. [17] conducted a systematic study on the formulation of semi-flexible grouting materials using conventional constituents, identifying an optimal mix ratio of the water–cement ratio: cement—fly ash—mineral powder = 0.58:1:0.1:0.1. Furthermore, advanced additives such as nanomaterials, ethylene vinyl acetate (EVA), and early-strength agents have been implemented to augment the performance characteristics of grouting materials [18,19,20,21,22]. Existing research on semi-flexible pavement (SFP) has predominantly concentrated on the characterization of porous asphalt mixtures and grouting materials, while the comprehensive performance evaluation of SFP itself remains a critical research focus. Extensive studies have confirmed that SFP exhibits superior Marshall stability, compressive strength, and tensile strength compared to conventional asphalt mixtures [17,23,24,25]. Xiong et al. investigated temperature and loading effects on SFP fracture behavior through semi-circular bending tensile tests [26], while Gong and Yang explored the influence of asphalt binder types on the fatigue characteristics of SFP materials [27,28]. Additionally, Zarei et al. evaluated the fatigue performance of SFP utilizing a cement–asphalt emulsion as the grouting medium [29].

However, limited attention has been paid to the full temperature-domain crack resistance of SFP materials and their structural crack resistance mechanisms within pavement systems. To address this knowledge gap, this study conducts a comparative analysis of crack resistance between SFP and SMA-13 asphalt mixtures across varying temperature conditions. Furthermore, the layered elastic system mechanics framework is employed to investigate the structural crack resistance enhancement mechanisms of SFP within pavement configurations.

## 2. Research Objectives and Methods

This study is designed to achieve the following research objectives:

1. Assess the crack resistance of semi-flexible pavement (SFP) materials through low-temperature beam bending tests, semi-circular bending (SCB) tensile tests, and indirect tensile tests.
2. Investigate the structural improvement effects of SFP on pavement crack resistance.

**Figure 1:**
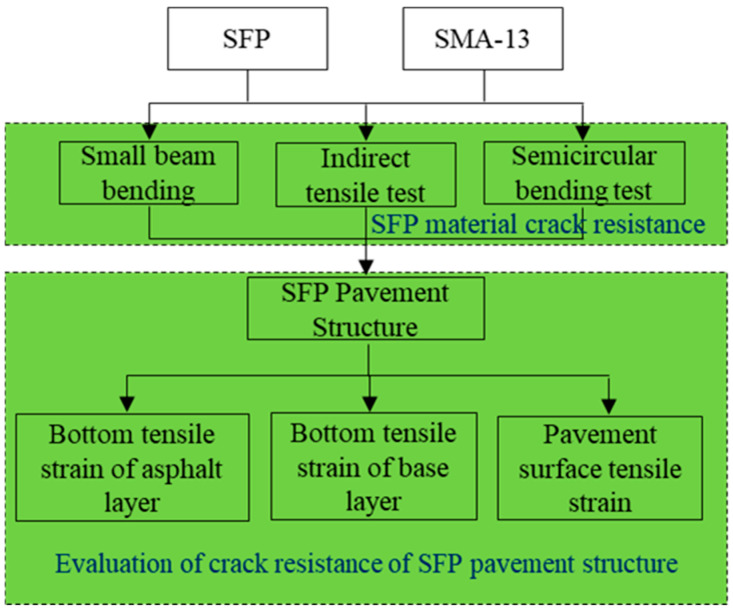
Research methodology.

The methodology framework is illustrated in Figure 1. First, SMA-13 and SFP specimens were prepared using identical aggregates and an SBS-modified asphalt binder. Subsequently, the crack resistance of the SFP was evaluated through comparative testing with SMA-13 under varied temperature conditions: low-temperature beam bending tests at −10 °C, semi-circular bending tensile tests at −10 °C, 20 °C, and 50 °C, and indirect tensile tests at 20 °C and 50 °C. Finally, the crack resistance performance of SFP-integrated pavement structures was analyzed using a layered elastic system mechanics approach. Critical mechanical responses, including surface tensile strain and bottom tensile strain of the asphalt layer, were quantified under vertical and horizontal loading conditions across different temperatures to evaluate structural crack resistance enhancement.

## 3. Materials and Tests

### 3.1. Materials

(1) Asphalt binder

SBS-modified asphalt was used for asphalt mixture fabrication in the present paper, and its basic properties are listed in Table 2.

(2) Aggregate

A basalt coarse aggregate and limestone mineral powder were used in the porous asphalt mixture and SMA-13. Their basic properties were tested and are presented in Table 3.

(3) Porous asphalt mixture and SMA-13

The porous asphalt mixtures and SMA-13 were formulated using identical asphalt binder and aggregate materials. Two graded porous asphalt mixtures, SFAC-13 and SFAC-16 [30], were selected for this study, with their matrix skeleton design gradations detailed in Table 4. The optimal asphalt contents for SFAC-13 and SFAC-16 were determined as 3.36% and 3.31%, respectively, yielding porosities of 23.5% and 24.1%. The Cantabro abrasion loss values measured 10.2% and 11.3% for SFAC-13 and SFAC-16, respectively. During porous asphalt mixture preparation, the asphalt binder was maintained at 175 °C. Marshall specimens were fabricated with dimensions of 101 mm diameter × 63.5 mm height, while gyratory-compacted specimens measured 150 mm diameter × 150 mm height. Six standardized specimens were prepared for each experimental group. The design gradation [31] of SMA-13 is presented in Table 4, with an optimum asphalt content of 5.8% determined through Marshall testing procedures.

(4) Grouting materials and SFP

The grouting material was formulated according to the following mix proportion: (rapid-hardening cement–Grade 42.5 cement = 0.15:0.85)–mineral powder–expansive agent–water-reducing agent–flocculant = 1:0.2:0.04:0.001:0.005 (by mass ratio). The grout was prepared by blending water with the dry grouting materials at a water-to-grouting-material ratio of 0.4 (by weight). The mixture was homogenized using a high-speed mixer operating at 5000 rpm for 2 min. The fundamental physicochemical properties of the grouting materials are summarized in Table 5.

**Figure 2:**
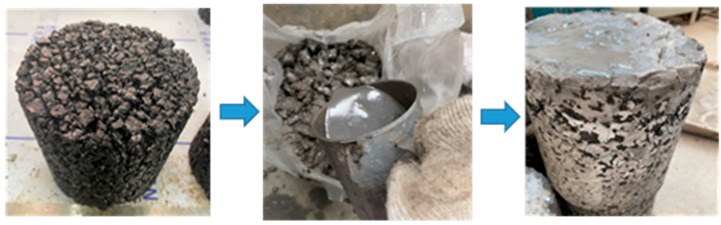
Grouting process of SFP.

The gradations of SFAC-13 and SFAC-16 were selected for the preparation of semi-flexible pavement (SFP) specimens by infusing grout materials into the SFAC-13 and SFAC-16 matrices. Following the fabrication of the porous asphalt mixtures, the bottom and peripheral surfaces of the specimens were sealed with plastic bags and adhesive tape to prevent grout leakage. The grout was subsequently injected into the porous structure. Upon solidification of the grouting material, the sealing materials were carefully removed. A schematic representation of the SFP grouting process is illustrated in Figure 2.

### 3.2. Tests

(1) Low-temperature beam test

The bending creep test serves as one of the effective methodologies for assessing the low-temperature performance characteristics of asphalt mixtures [32,33]. This study employed plate specimens of semi-flexible pavement (SFP) with dimensions of 300 mm × 300 mm × 50 mm, which were subsequently cut into beam specimens measuring 250 mm × 35 mm × 30 mm. The low-temperature beam bending test was conducted after conditioning the beams in a temperature-controlled chamber at −10 °C for 4 h. The test parameters included a controlled temperature of −10 °C and a loading rate of 2 mm/min. The flexural tensile strength (*R_B_*), maximum flexural tensile strain (*ε_B_*), and bending stiffness modulus (*S_B_*) at failure were calculated using the following equations:

(1) $$\begin{array}{l} R_{B}=\frac{3\times L\times P_{B}}{2\times b\times h^{2}}\\ \varepsilon_{B}=\frac{6\times h\times d}{L^{2}}\\ S_{B}=\frac{R_{B}}{\varepsilon_{B}} \end{array}$$

where *R_B_* is flexural tensile strength of the test piece at failure (MPa), *ε_B_* is maximum flexural tensile strain of the specimen at failure (με), *S_B_* is the bending stiffness modulus at the time of specimen failure (MPa), b is the width of the interview piece across the interruption (mm), *h* is the height of the interview piece across the interruption (mm), L is span of the test piece (mm), *P_B_* is the maximum load when the test piece is failure (N), and *d* is midspan deflection when the specimen is failure (mm).

(2) Indirect tensile test

**Figure 3:**
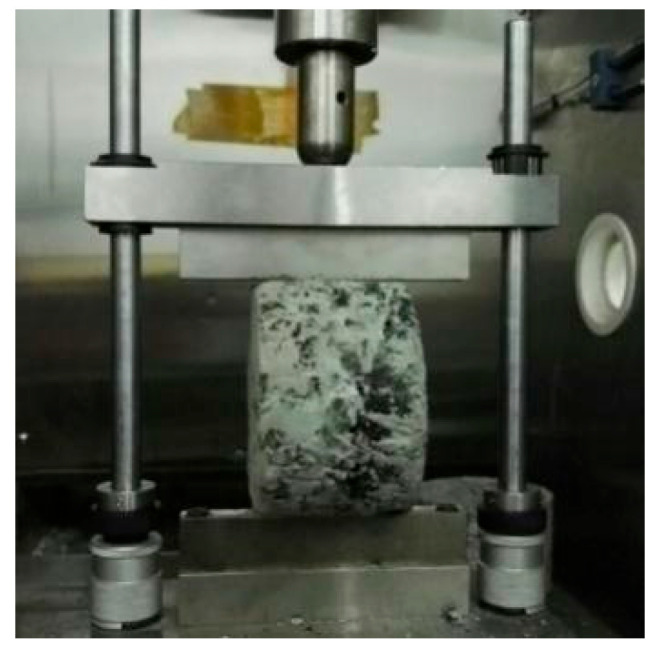
IDT test apparatus.

The indirect tensile test (IDT) [34] was conducted using Marshall specimens with dimensions of 100 mm in diameter and 63.5 mm in height. Testing was performed in accordance with ASTM D6931 [35], wherein a vertical diametral load was applied to specimens cured for 3 d, 7 d, and 28 d. The tests were executed at controlled temperatures of 20 °C and 50 °C with a constant loading rate of 50 mm/min. The experimental configuration and loading procedure are schematically illustrated in Figure 3.

(3) Semi-circular bending (SCB) tensile test

**Figure 4:**
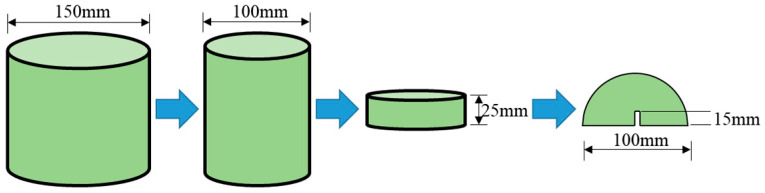
Specimen dimensions of the SCB test.

**Figure 5:**
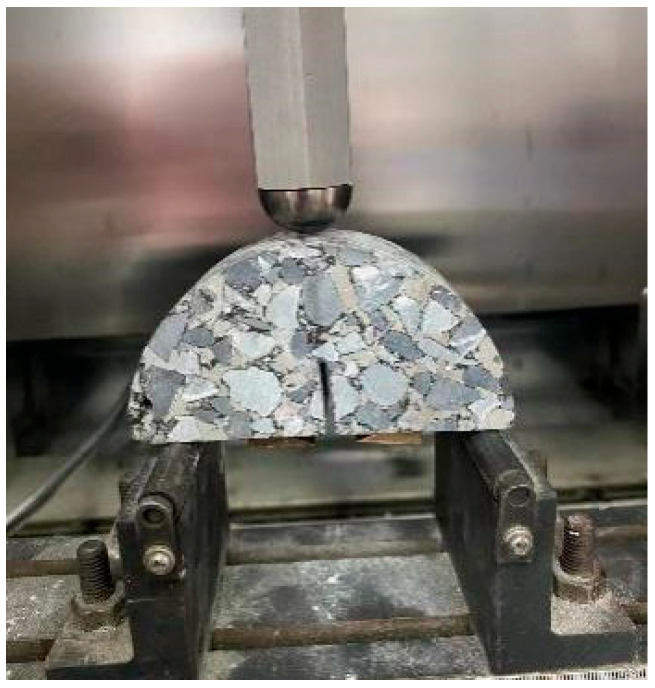
SCB test.

**Figure 6:**
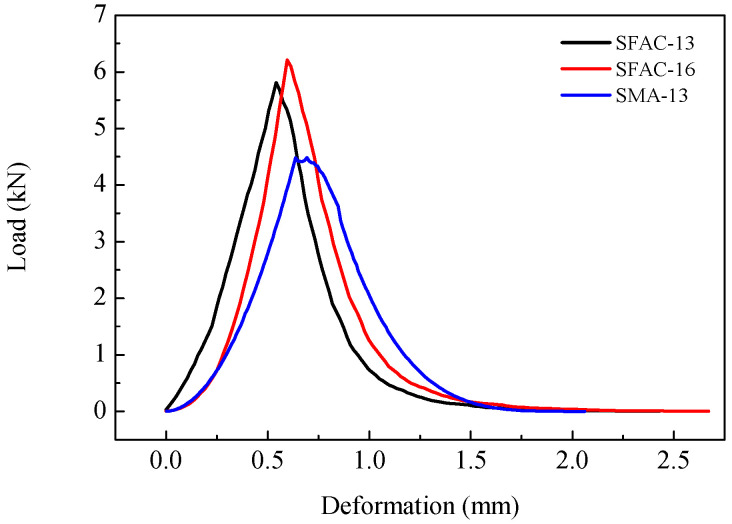
Typical load–displacement curve.

**Figure 7:**
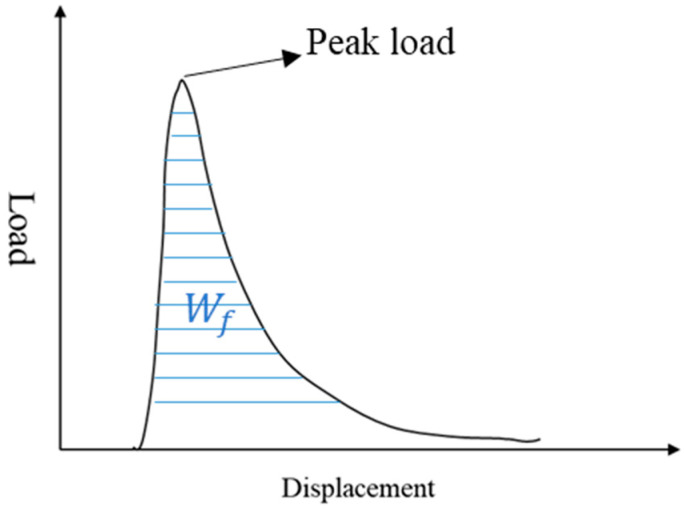
Typical displacement–force curve of SCB test.

The semi-circular bend (SCB) test, originally developed to assess the fracture toughness of brittle materials, has been adapted by pavement researchers to evaluate the fracture characteristics of asphalt mixtures [36,37]. SCB specimens were fabricated from gyratory-compacted asphalt mixtures and machined into half-moon geometries with dimensions of 100 mm diameter, 50 mm height, and 25 mm thickness, featuring a 15 mm straight-edge notch (as shown in Figure 4). The testing configuration and procedure are illustrated in Figure 5 and Figure 6. Key fracture parameters, including tensile strength and fracture energy (visualized in Figure 7), were calculated using Equations (2) and (3) [38,39,40].

(2) $$S=\frac{4.976F}{BD}$$

where *S* is the tensile strength (MPa), *F* is the value of the peak load (N), *B* is the height of the specimen (mm), and *D* is the diameter of the specimen (mm).

(3) $$G_{f}=\frac{W_{f}}{BD}$$

where $G_{f}$ is the fracture energy and $W_{f}$ is the fracture work.

(4) Dynamic Modulus Testing

The dynamic modulus has been widely adopted as a critical structural design parameter for asphalt pavements in numerous countries [41,42,43]. Specimens with dimensions of 150 mm in diameter and 170 mm in height were fabricated using a Superpave Gyratory Compactor (SGC). Following grouting and curing, cylindrical cores were extracted for dynamic modulus testing.

(5) Analytical Methodology

**Figure 8:**
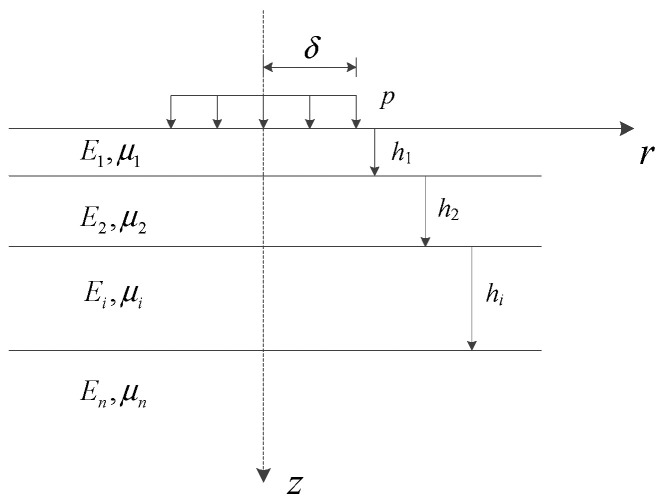
Multilayered elastic system.

A layered elastic mechanics (LEM) framework incorporating interlayer adhesion was employed for structural analysis and the model was shown in Figure 8. Interlayer stress coefficients were computed via the coefficient recurrence method, while pavement surface mechanical responses were derived using the surface residual method. The analytical program (PADS), previously validated in reference [44], was utilized to evaluate cracking behavior in SFP pavement structures. A uniformly distributed vertical load with a circular contact area (radius *r* = 10.65 cm, pressure *p* = 0.7 MPa) was applied in the computational model.

## 4. SFP Material Crack Resistance

### 4.1. Low-Temperature Beam Test Result

**Figure 9:**
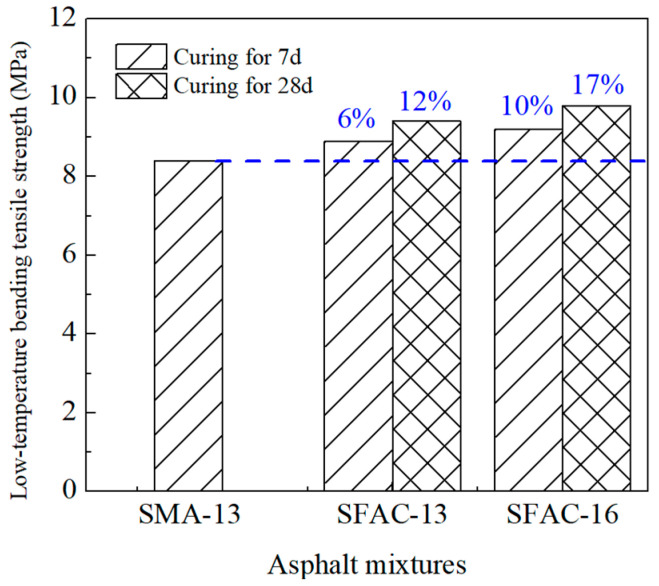
Low-temperature bending tensile strength of three asphalt mixtures.

**Figure 10:**
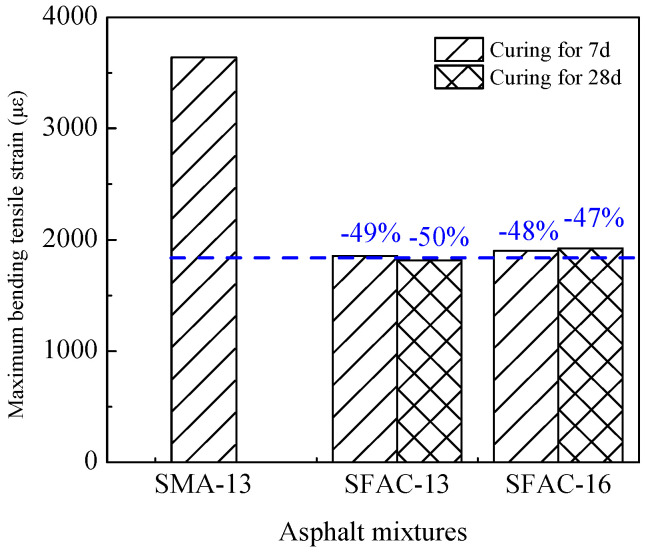
Maximum bending tensile strain of three asphalt mixtures.

The low-temperature beam bending test results of SFP materials and SMA-13 are presented in Table 6 and Figure 9 and Figure 10. As demonstrated in Table 6, the flexural tensile strength of SFP materials exhibits an increasing trend with extended curing durations. Both the SFAC-13 and SFAC-16 materials display comparable characteristics in terms of flexural tensile strength, flexural tensile strain, and flexural modulus, primarily owing to their similar porosity levels and grouting material quantities. Figure 9 and Figure 10 reveal that the semi-flexible pavement materials achieve 10–20% higher flexural tensile strength compared to SMA-13, while their flexural tensile strain is reduced to approximately half of that observed in SMA-13. This phenomenon indicates that the incorporation of cementitious grout significantly enhances the low-temperature brittleness of SFP materials. Although low-temperature flexural tensile strain has traditionally served as a critical indicator for evaluating the low-temperature performance of asphalt mixtures, the comprehensive analysis of SFP materials necessitates a synergistic consideration of both strength evolution and deformation resistance mechanisms.

### 4.2. Indirect Tensile Test Result

**Figure 11:**
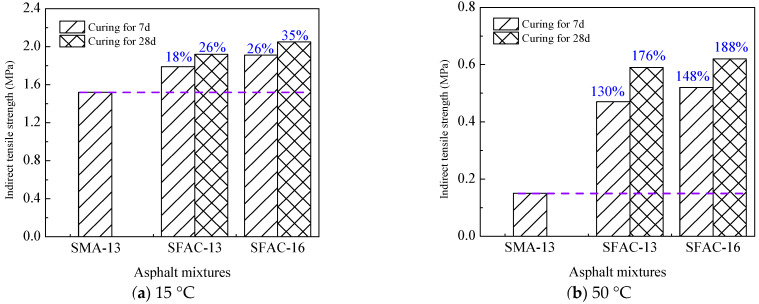
IDT test results.

The indirect tensile test, as illustrated in Figure 3, was performed to determine the peak loads of SFPs and SMA-13 under varying curing durations and temperatures. The calculated indirect tensile strength results are summarized in Table 7 and depicted in Figure 11. For SFP materials, the indirect tensile strength exhibits a progressive enhancement with prolonged curing time, with SFAC-13 and SFAC-16 demonstrating comparable strength values. Notably, the indirect tensile strength of SFPs at 15 °C exceeds that of SMA-13 by 20–35%, while at 50 °C, this difference amplifies to 130–190%. These findings indicate that the SFP materials not only exhibit superior deformation resistance [45,46] but also demonstrate exceptional high-temperature crack resistance. This performance advantage can be attributed to the temperature-independent strength characteristics of the grouting material and the robust interfacial bonding between the grouting material and asphalt matrix, which synergistically ensure enhanced mechanical integrity under elevated thermal conditions.

### 4.3. Semi-Circular Bending (SCB) Tensile Test Result

The semi-circular bend (SCB) test and representative load–displacement curves are illustrated in Figure 5 and Figure 6, respectively. As evident from Figure 6, the load progressively increases with deformation until reaching peak load, after which it exhibits a sustained decline due to cohesive zone effects. Both SFAC-13 and SFAC-16 demonstrate higher peak loads compared to SMA-13. Furthermore, the slopes of their load–deformation curves, both pre- and post-peak, exceed those of SMA-13. Notably, SMA-13 exhibits slower load variations near peak load relative to SFAC-13 and SFAC-16. These observations indicate that the incorporation of cement-based grouting materials enhances the strength and stiffness of SFP materials beyond SMA-13, though their cohesive performance in fracture zones remains inferior to SMA-13.

**Figure 12:**
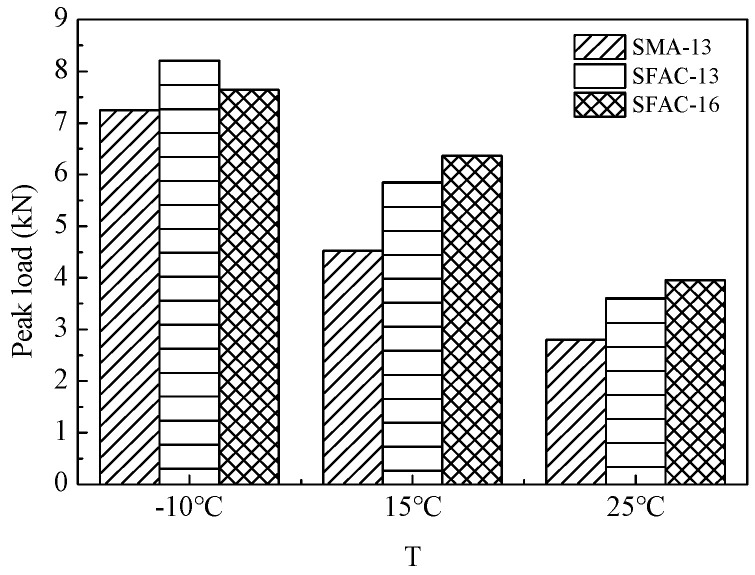
Peak load.

**Figure 13:**
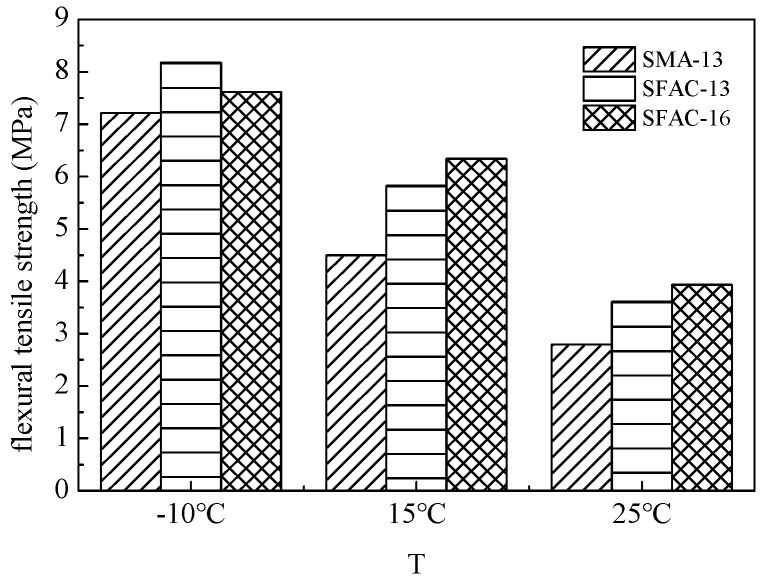
Flexural tensile strength.

**Figure 14:**
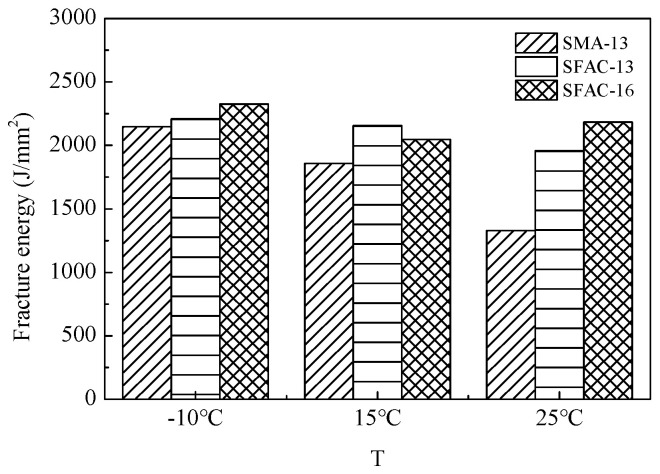
Fracture energy of three types of asphalt mixtures.

The SCB test results, summarized in Table 8 and Figure 12, Figure 13 and Figure 14, reveal comparable low-temperature performance between the SMA-13 and SFP materials (SFAC-13/SFAC-16) in terms of peak load, flexural tensile strength, and fracture energy. However, this performance gap widens significantly with temperature elevation. At 15 °C, the semi-flexible materials exhibit 1.3-fold higher peak load and flexural tensile strength than SMA-13, accompanied by a 1.15-times greater fracture energy. This disparity amplifies at 25 °C, where the semi-flexible materials demonstrate 1.4-fold increases in peak load and flexural tensile strength, coupled with 1.5-fold enhancement in fracture energy relative to SMA-13. These findings suggest equivalent low-temperature crack resistance between the SFP materials and SMA-13, while the SFP materials exhibit superior crack resistance at ambient temperatures.

## 5. SFP Pavement Structure Crack Resistance

**Figure 15:**
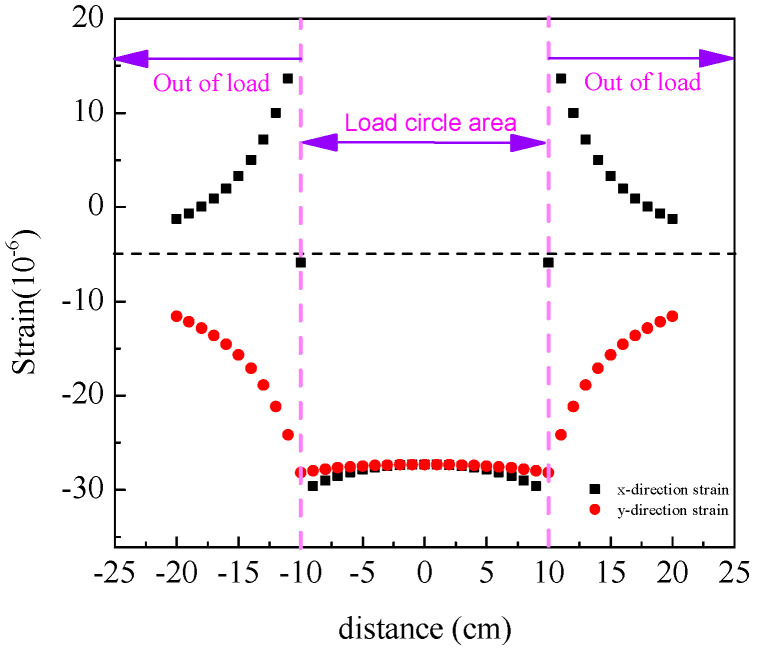
Strain distribution on pavement surface.

**Figure 16:**
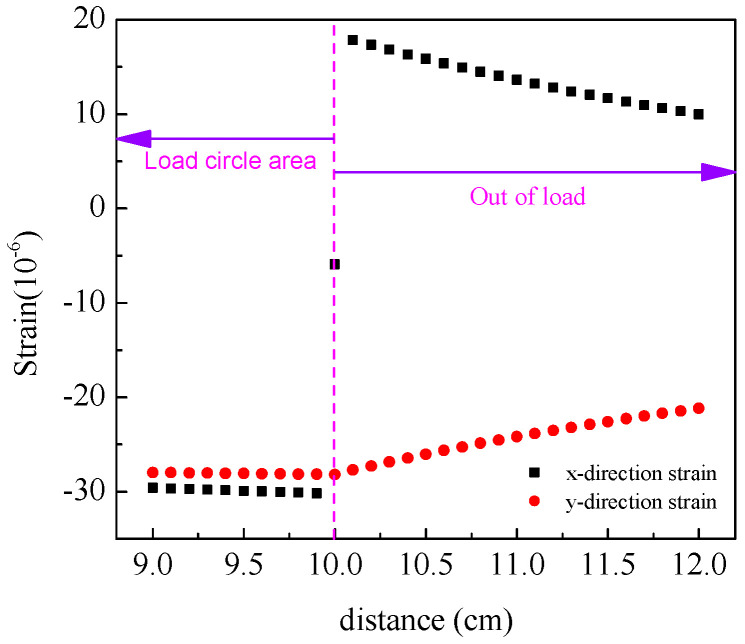
Strain distribution at the edge of the load.

Layered elastic mechanics serves as the mechanical analysis model for asphalt pavement, with computational implementation achieved detailed in part (5) of Section 3.2. The layered elastic system serves as a mechanical model for asphalt pavement structures. By conceptualizing each pavement structural layer as isotropic homogeneous elastic bodies, this model enables the analytical investigation of structural mechanical responses under varying temperature conditions and different interlayer bonding states through the application of vehicular loading. Contemporary research has identified top–down cracking, driven by tensile strain at the pavement surface [47,48,49], as a distinct cracking mode in asphalt pavements, contrasting with traditional bottom–up fatigue cracking. The assessment of cracking resistance constitutes a critical component in pavement design [35,37]. Conventionally, the maximum tensile strain at the asphalt layer base is adopted as the analytical index for fatigue cracking evaluation, typically assumed to occur at the layer’s base under the load center. Conversely, for top–down cracking analysis, the maximum tensile strain at the pavement surface, predominantly localized near the tire load periphery [50], serves as the evaluation criterion. Figure 15 and Figure 16 illustrate the strain distributions at the pavement surface and load edge, respectively. As depicted in Figure 15, the tensile strain in the x-direction at the load periphery contributes to top–down crack initiation, while compressive strains in both the y-direction and intra-load x-direction exhibit negligible distress potential. Figure 16 further demonstrates an exponential decay of surface tensile strain with increasing distance from the load edge. Consequently, the maximum strain value at the load edge emerges as the principal analytical parameter for quantifying top–down cracking susceptibility.

**Figure 17:**
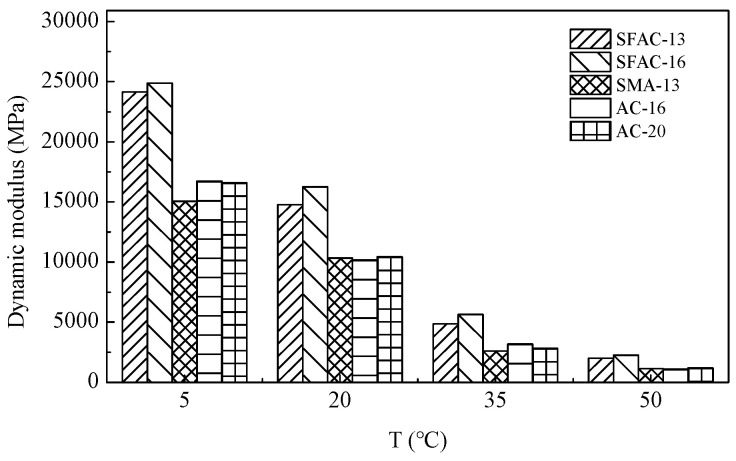
Dynamic modulus of five asphalt mixtures at different temperatures.

In this investigation, layered elastic mechanics (LEM) was employed to assess the crack resistance of semi-flexible pavement (SFP) structures, with the dynamic modulus serving as the primary material parameter. The dynamic modulus values at a frequency of 10 Hz under varying temperatures were determined using a universal testing machine (UTM), with experimental results tabulated in Table 9 and graphically represented in Figure 17. As demonstrated in Table 9 and Figure 17, the incorporation of cement-based grouting materials significantly enhances the dynamic modulus of SFP materials compared to conventional asphalt mixtures. This elevation in the dynamic modulus underscores the improved stiffness and load-bearing capacity imparted by the grouted composite system.

To assess the crack resistance of semi-flexible pavement (SFP) structures, a semi-rigid base pavement configuration—representing the most widely utilized pavement structure in China (as detailed in Table 10)—was adopted as the analytical framework. Multiple interlayer adhesion conditions between the ATB-25 layer and cement-stabilized macadam base were investigated, including fully bonded, 100 MPa/cm, 500 MPa/cm, and debonded scenarios. Two distinct SFP structural configurations were evaluated:

Case 1: Single-layer SFP structure, substituting the SMA-13 surface layer in Table 10 with SFAC-13.
Case 2: Double-layer SFP structure, replacing both the SMA-13 and AC-16 layers in Table 10 with SFAC-13 and SFAC-16, respectively.

This comparative analysis enables the systematic evaluation of crack resistance enhancement achieved through SFP material integration under varying structural and interfacial bonding conditions.

**Figure 18:**
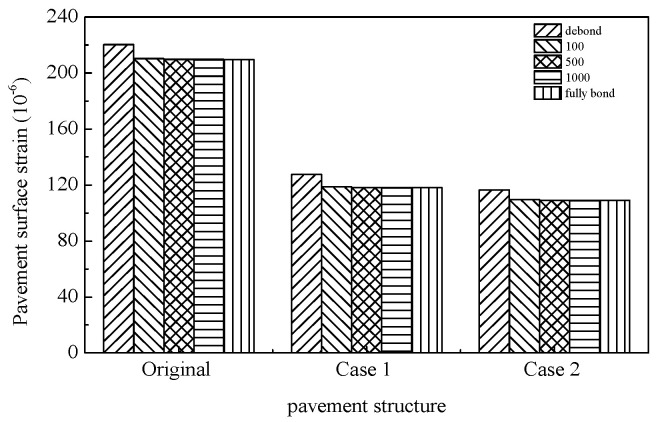
Surface tensile strain of different pavement structures (50 °C).

**Figure 19:**
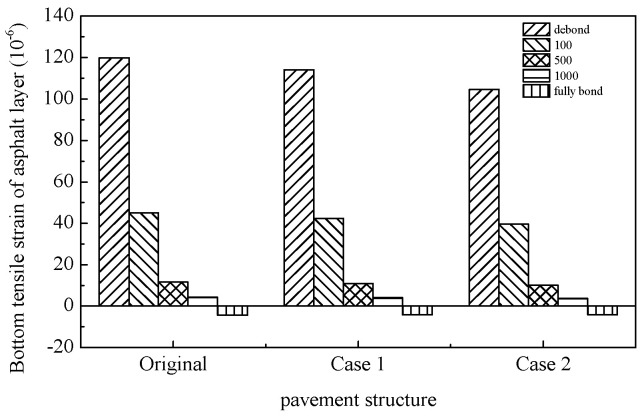
Tensile strain of asphalt layer bottom with different pavement structures (50 °C).

**Figure 20:**
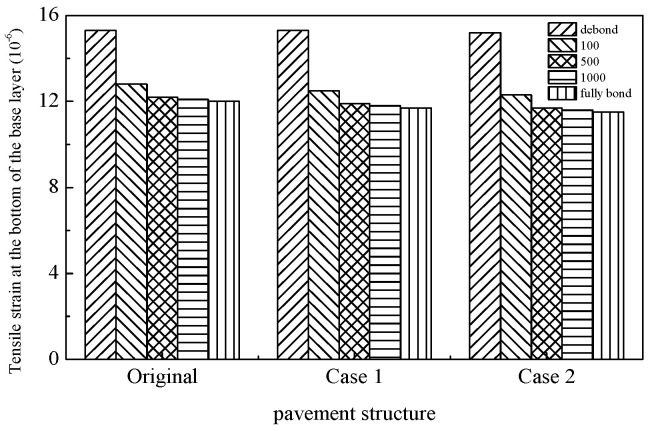
Tensile strain at bottom of base layer with different pavement structures (50 °C).

The tensile strains at the pavement surface, asphalt mixture layer base, and base layer—commonly utilized to evaluate top–down cracking, asphalt layer fatigue cracking, and base layer fatigue cracking, respectively—were calculated as summarized in Table 11, Table 12 and Table 13. The results indicate that both pavement surface tensile strain and tensile strain at the base of the asphalt layer/base layer exhibit a continuous increase with rising temperatures, suggesting that elevated thermal conditions exacerbate susceptibility to top–down and fatigue cracking in asphalt pavements. Concurrently, the interlayer bonding state between the base and asphalt mixture layers exerts differential influences on pavement cracking mechanisms. Under weakened interlayer bonding conditions, amplified tensile strains at the asphalt layer base and base layer significantly elevate risks of fatigue cracking in these structural components. Notably, at high temperatures, compromised interlayer bonding induces a transition of the asphalt layer from a compressive to tensile stress state. A temperature-dependent relationship is observed regarding the impact of interlayer bonding on pavement surface tensile strain. At low temperatures, surface tensile strain escalates with increasing interlayer bonding coefficients. Under moderate temperatures, this strain initially decreases before rising with higher bonding coefficients, while at elevated temperatures, it demonstrates an inverse correlation with bonding coefficients. An analysis of Table 11, Table 12 and Table 13 and Figure 18, Figure 19 and Figure 20 reveals that the SFP pavement structures exhibit limited enhancements in asphalt layer and base layer fatigue resistance. The single-layer SFP configuration (Case 1) achieves a 50% reduction in pavement surface tensile strain compared to the conventional structures, whereas the double-layer SFP system (Case 2) shows negligible improvement. These findings suggest that semi-flexible materials are strategically applicable as surface layers in road sections prone to severe top–down cracking (e.g., intersections and bus stops), while dual-layer SFP implementations may be prioritized for areas experiencing concurrent rutting and surface cracking distress. This stratified application approach optimizes material functionality based on predominant failure modes.

## 6. Conclusions

This study systematically evaluated the crack resistance of semi-flexible pavement (SFP) materials and structures through low-temperature beam bending tests, indirect tensile tests, semi-circular bend (SCB) tests, and layered elastic mechanics (LEM) analysis. The principal findings are summarized as follows:

(1) A comparative analysis of low-temperature flexural tensile strength, indirect tensile strength, and fracture energy between the SFP materials and SMA-13 reveals that the SFP materials exhibit superior crack resistance at ambient and elevated temperatures. However, at low temperatures, while the SFP materials demonstrate comparable flexural strength and fracture energy to SMA-13, their significantly reduced flexural tensile strain indicates limitations in low-temperature ductility. These results suggest that low-temperature flexural tensile strain alone cannot holistically assess the low-temperature crack resistance of SFP materials.
(2) Compromised interlayer bonding between the surface and base layers substantially exacerbates fatigue cracking at the asphalt layer base and semi-rigid base layer yet exerts minimal influence on surface-initiated cracking mechanisms.
(3) The implementation of semi-flexible materials as surface layers effectively enhances pavement surface crack resistance but demonstrates negligible improvement in fatigue cracking resistance for both the asphalt layers and semi-rigid base layers.
(4) Double-layer SFP configurations exhibit comparable surface crack resistance to single-layer SFP systems. Strategic application recommendations are proposed. Single-layer SFP materials are advised for road sections with moderate rutting severity (e.g., intersections and long longitudinal slopes). Double-layer SFP materials are recommended for high-stress zones experiencing concurrent severe rutting and surface cracking (e.g., heavy-duty traffic lanes).

It should be noted that this paper only focuses on material properties and theoretical analysis, and the actual effects need to be verified through SFP pavement experiments. During the recycling process, the material should be separated into asphalt mixture and cement grout components through integrated image analysis and mechanical processing for subsequent recycling treatment.

## Figures and Tables

**Table 1:** Technical requirements for grouting materials [30].

Technical Index	Requirements
Mobility (s)	10~14
Shrinkage rate of 7 d (%)	<0.3
Bleeding ratio (3 h)	<3
Compressive strength of 7 d (MPa)	10~30
Flexural strength 7 d (MPa)	>2

**Table 2:** General characteristics of asphalt.

Asphalt		SBS Asphalt
Penetration, 0.1 mm	30 °C	97.7
	25 °C	74.1
	15 °C	26.2
Ductility (cm)		55.2 (5 °C)
Softening point (°C)		87.5

**Table 3:** Properties of aggregate.

Properties	Unit	Sieve Size (mm)						Mineral Powder
		16~19	13.2~16	9.5~13.2	4.75~9.5	2.36~4.75	0~2.36	
Apparent density	g/cm^3^	2.954	2.942	2.935	2.857	2.845	2.847	2.686
Bulk density	g/cm^3^	2.862	2.875	2.863	2.824	2.758	—	—
Water absorption	/%	1.14	1.45	1.23	1.75	1.23	—	—

**Table 4:** Grading composition of three asphalt mixtures.

Types of Mixtures	Sieve Passing Rate (%)											Optimum Asphalt Content (%)
	19	16	13.2	9.5	4.75	2.36	1.18	0.6	0.3	0.15	0.075	
SFAC-13	100	100	95	53.5	12	10	8.5	8	6.5	6	4	3.36
SFAC-16	100	95	85	50	15	12	9	8	7	5	4	3.31
SMA-13	100	100	97	59.5	26.5	21.3	17.9	14.7	12.4	11.2	9.6	5.8

**Table 5:** Basic properties of grouting materials.

Test Items	Water Cement Ratio	Fluidity (s)	Flexural Strength (MPa)		Compressive Strength (MPa)	
			4 h	7 d	4 h	7 d
GM	0.40	12.9	2.3	5.8	18	29

**Table 6:** Low-temperature bending tensile properties of SFP.

Asphalt Mixtures	Low-Temperature Bending Tensile Strength (MPa)	Maximum Bending Tensile Strain (10^−6^)	Bending Modulus (MPa)
SMA-13	8.4	3642	2306
SFAC-13 (7 d)	8.9	1856	4795
SFAC-13 (28 d)	9.4	1816	5176
SFAC-16 (7 d)	9.2	1902	4837
SFAC-16 (28 d)	9.8	1923	5096

**Table 7:** Indirect tensile strength results of SMA-13 and SFPs (MPa).

Temperatures	SMA-13	SFAC-13		SFAC-16	
		7 d	28 d	7 d	28 d
15 °C	1.52	1.79	1.92	1.91	2.05
50 °C	0.15	0.47	0.59	0.52	0.62

**Table 8:** SCB test results of three types of asphalt mixtures.

Temperature (°C)	Parameter	SMA-13	SFAC-13	SFAC-16
−10	Peak load (kN)	7.25	8.21	7.65
	Flexural tensile strength (MPa)	7.22	8.17	7.61
	Fracture energy (J/mm^2^)	2147	2209	2325
15	Peak load (kN)	4.52	5.85	6.37
	Flexural tensile strength (MPa)	4.50	5.82	6.34
	Fracture energy (J/mm^2^)	1857	2156	2048
25	Peak load (kN)	2.80	3.63	3.95
	Flexural tensile strength (MPa)	2.79	3.61	3.93
	Fracture energy (J/mm^2^)	1328	1956	2185

**Table 9:** Dynamic modulus of five asphalt mixtures at different temperatures.

Asphalt Mixtures	Dynamic Modulus (MPa)			
	5 °C	20 °C	35 °C	50 °C
SFAC-13	24,128	14,782	4862	2003
SFAC-16	24,892	16,254	5642	2244
SMA-13	15,062	10,333	2602	1144
AC-16	16,736	10,180	3182	1090
AC-20	16,611	10,418	2831	1195

**Table 10:** Semi-rigid base pavement structure.

Materials	Thickness (cm)	Modulus (MPa)	Poisson’s Ratio
SMA-13	5	Determined by temperature	0.25
AC-16	6	Determined by temperature	0.25
AC-20	7	Determined by temperature	0.25
Cement-stabilized macadam	20	16,000	0.25
Cement-stabilized macadam	16	8500	0.25
Graded aggregate	15	320	0.3
Subgrade	—	70	0.35

**Table 11:** Tensile strain at different positions (5 °C).

Mechanical Response	Pavement Structure	Interlayer Bonding Condition (MPa/cm)				
		Debond	100	500	1000	Fully Bond
Pavement surface tensile strain	Original	7.9	8.2	8.8	9.1	9.3
	Case 1	3.1	3.4	3.9	4.1	4.4
	Case 2	2.6	3.2	3.8	3.9	4.2
Tensile strain at the bottom of asphalt layer	Original	19.8	12.8	8.6	7.1	4.4
	Case 1	19.3	12.5	8.5	7.0	4.5
	Case 2	18.7	12.23	8.4	7.0	4.6
Tensile strain at the bottom of the base layer	Original	13.2	10.1	8.7	8.4	7.9
	Case 1	12.8	9.6	8.3	7.9	7.5
	Case 2	12.6	9.5	8.1	7.8	7.3

**Table 12:** Tensile strain at different positions (20 °C).

Mechanical Response	Pavement Structure	Interlayer Bonding Condition (MPa/cm)				
		Debond	100	500	1000	Fully Bond
Pavement surface tensile strain	Original	16.6	15.6	15.9	16.1	16.3
	Case 1	10.2	9.4	9.7	9.8	10.0
	Case 2	9.3	8.8	9.3	9.4	9.7
Tensile strain at the bottom of asphalt layer	Original	24.1	14.9	8.5	6.2	1.9
	Case 1	23.7	14.6	8.4	6.2	2.1
	Case 2	22.9	14.3	8.4	6.2	2.3
Tensile strain at the bottom of the base layer	Original	13.6	10.5	9.11	8.7	8.3
	Case 1	13.4	10.1	8.8	8.4	7.9
	Case 2	13.2	9.9	8.5	8.2	7.7

**Table 13:** Tensile strain at different positions (50 °C).

Mechanical Response	Pavement Structure	Interlayer Bonding Condition (MPa/cm)				
		Debond	100	500	1000	Fully Bond
Pavement surface tensile strain	Original	220.4	210.2	209.7	209.7	209.6
	Case 1	127.6	118.7	118.3	118.3	118.3
	Case 2	116.61	109.4	109.2	109.2	109.2
Tensile strain at the bottom of asphalt layer	Original	119.8	45.0	11.6	4.3	−4.4
	Case 1	114.1	42.4	10.9	4.0	−4.2
	Case 2	104.5	39.6	10.1	3.7	−4.1
Tensile strain at the bottom of the base layer	Original	15.3	12.8	12.2	12.1	12.0
	Case 1	15.3	12.5	11.9	11.8	11.7
	Case 2	15.2	12.3	11.7	11.6	11.5

## Data Availability

The original contributions presented in this study are included in the article. Further inquiries can be directed to the corresponding authors.
